# Cloning and expression of porcine Colony Stimulating Factor-1 (CSF-1) and Colony Stimulating Factor-1 Receptor (CSF-1R) and analysis of the species specificity of stimulation by CSF-1 and Interleukin 34

**DOI:** 10.1016/j.cyto.2012.08.008

**Published:** 2012-12

**Authors:** Deborah J. Gow, Valerie Garceau, Ronan Kapetanovic, David P. Sester, Greg J. Fici, John A. Shelly, Thomas L. Wilson, David A. Hume

**Affiliations:** a The Roslin Institute, Royal (Dick) School of Veterinary Studies, University of Edinburgh, Easter Bush, Midlothian EH25 9RG, Scotland, UK; b Pfizer Animal Health, 7000 Portage Road, Kalamazoo, MI 49001, United States

**Keywords:** Macrophage, Ba/F3 cells, Bone marrow, Hematopoiesis, Species specificity

## Abstract

Macrophage Colony Stimulating Factor (CSF-1) controls the survival, differentiation and proliferation of cells of the mononuclear phagocyte system. A second ligand for the CSF-1R, Interleukin 34 (IL-34), has been described, but its physiological role is not yet known. The domestic pig provides an alternative to traditional rodent models for evaluating potential therapeutic applications of CSF-1R agonists and antagonists. To enable such studies, we cloned and expressed active pig CSF-1. To provide a bioassay, pig CSF-1R was expressed in the factor-dependent Ba/F3 cell line. On this transfected cell line, recombinant porcine CSF-1 and human CSF-1 had identical activity. Mouse CSF-1 does not interact with the human CSF-1 receptor but was active on pig. By contrast, porcine CSF-1 was active on mouse, human, cat and dog cells. IL-34 was previously shown to be species-specific, with mouse and human proteins demonstrating limited cross-species activity. The pig CSF-1R was equally responsive to both mouse and human IL-34. Based upon the published crystal structures of CSF-1/CSF-1R and IL34/CSF-1R complexes, we discuss the molecular basis for the species specificity.

## Highlights

► The active region of porcine CSF-1 and full length CSF-1R have been cloned. ► Biological activity of porcine CSF-1 has been demonstrated. ► The cross species reactivity of IL-34 has been investigated. ► Both mouse and human IL-34 are biologically active on the pig CSF-1R. ► CSF-1 and IL-34 structure analysis of species-specific activity has been performed.

## 1 Introduction

Macrophage Colony-Stimulating Factor (CSF-1) is required for the proliferation, differentiation and survival of cells of the mononuclear phagocyte lineage [1–3]. CSF-1 signals through a protein tyrosine kinase receptor, CSF-1R, which in adults is restricted in its expression to myeloid cells [4]. The three-dimensional structure of the CSF-1/CSF-1R complex has been reported [5]. Cells of mesenchymal lineages (fibroblasts, osteoblasts, myoblasts, adipocytes, endothelial cells) produce CSF-1 constitutively, and the protein is detected in the circulation at biologically active concentrations [6,7]. In keeping with these cellular sources, almost every tissue in the body can produce CSF-1 [8]. Elevated levels in uterus, endometrium, oviducts and conceptus suggest that CSF-1 functions in foetal growth and development, consistent with expression of the receptor in placental trophoblast cells [9–11].

In mammals, alternative splicing from the CSF-1 locus generates several isoforms. Garceau et al. [12] demonstrated that the two major mRNAs are also present in birds. All functional CSF-1 isoforms share a biologically-active 149 residue growth factor domain, making CSF-1 a member of the four helix bundle hemopoietin family [13]. This domain contains four intra-chain disulphide bridges, and forms a disulphide-linked dimer [14]. The avian protein lacks the inter-chain disulphide bond, but like the related stem cell factor, still forms functional homodimers [12]. The smallest protein product of the CSF-1 gene is a cell surface glycoprotein with a transmembrane region and a cytoplasmic tail. Larger isoforms include a glycoprotein and proteoglycan, containing a variable spacer domain which permits cleavage from the cell surface to generate the soluble cytokine [8,13,15].

Mice and rats with a homozygous mutation in their CSF-1 gene are deficient in biologically active CSF-1 (*op*/*op* mice and *tl*/*tl* rats) and born with congenital osteopetrosis, reduced tissue macrophage populations and multiple developmental abnormalities [1,2,16]. Expression of full-length CSF-1 transgene or cell surface CSF-1 can fully correct the *op*/*op* mice weight defects [17,18]. These findings suggest that the regulated expression of the distinct CSF-1 isoforms is biologically significant [16].

A second ligand for the CSF-1R, Interleukin 34 (IL-34), has been identified in humans and mice [19], perhaps explaining the more penetrant phenotype of a csf1r (−/−) mutation in mice compared to the *op*/*op* [20]. Recent solution of the crystal structure of hIL-34 confirmed the prediction [12] that it belongs to the short four helical cytokine family that includes stem cell factor and Flt3L as well as CSF-1 [19,21]. The two ligands, one receptor, relationship was found to be conserved in birds and a co-evolution analysis indicated that the two ligands probably bind to distinct regions of the receptor [12]. Solution of the crystal structure of IL-34 bound to CSF-1R indicated that although both IL-34 and CSF-1 bind to the CSF-1R D2 and D3 domains, no single interaction with CSF-1R is shared between IL-34 and CSF-1 [21,22]. In keeping with this view, antibodies have been produced which can block CSF-1, but not IL-34 binding to the receptor [23]. Nandi et al. [24] have reported non-overlapping distributions of the two ligands in developing brain, and implicated IL-34 in brain development. IL-34 deficiency in a knockout mouse appears to generate a specific deficiency in microglia and Langerhans cells [25].

Molecular tools targeting either CSF-1 or CSF-1R have proved very useful, allowing investigation of the myeloid population (macrophages/monocytes). Antibodies against the CSF-1R developed for the mouse and human ligands (CD115) are commonly used as markers to enable purification of blood monocytes in mice and humans [26,27]. Injection of recombinant CSF-1 into mice and humans increases monocyte and macrophage numbers [2,28]. Conversely, antibodies that block receptor binding can deplete a subset of monocytes and the majority of tissue macrophages [29]. For these reasons, CSF-1 and its receptor have been recognised as candidate drug targets in humans [30–33].

Much of our knowledge of CSF-1 biology derives from studies of rodents. We have reviewed elsewhere the evidence of a role for macrophages, and CSF-1, in the growth hormone/insulin like growth factor-1 axis [16]. However, we have also demonstrated that the responses of mouse and human macrophages to the addition of CSF-1 are quite different; in mice CSF-1 is associated with induction of genes involved in wound repair, such as urokinase plasminogen activator, whereas in humans, CSF-1 induces genes involved in cholesterol biosynthesis [34]. The domestic pig has many similarities with humans, especially in terms of innate immune responses [35]. In the current study, we aimed to express porcine CSF-1, and the CSF-1 receptor, and to develop a simple bioassay to enable the study of CSF-1 and IL-34 biology in this species.

## 2 Materials and methods

### 2.1 Cell culture and reagents

The Ba/F3 cell line, transfected Ba/F3 cells and primary bone marrow cells were cultured in RPMI 1640 medium (Sigma–Aldrich, Dorset, UK) containing 10% HI-FCS (Sigma), 2 mM l-glutamine (35050-61 Invitrogen Ltd., Paisley, UK), 100 μg/ml streptomycin, and 100 U/ml penicillin (15140 Invitrogen Ltd., Paisley, UK). Untransfected Ba/F3 cells were maintained in medium containing 10% conditioned medium from X63 Ag8-653 myeloma cells carrying an expression vector for IL-3 [36,37]. Unless otherwise stated, transfected Ba/F3 cells and primary bone marrow cells were maintained in medium with 10^4^ U/ml rh-CSF-1 (a gift from Chiron Corp., Emeryville, CA, USA). HEK293T cells (American Type Culture Collection, Manassas, VA, USA) were cultured in DMEM (Sigma–Aldrich, Dorset, UK) supplemented with 10% HI-FCS (Sigma), 2 mM l-glutamine (Invitrogen), 100 μg/ml streptomycin (Invitrogen), 100 U/ml penicillin and 0.1 mM nonessential amino acids (Invitrogen). All cell lines and primary bone marrow cells were incubated at 37 °C with 5% CO_2_.

### 2.2 Total RNA extraction

All procedures performed on these animals were in accordance with national regulations and established guidelines and were reviewed and approved by the Institutional Animal Care and Use Committee or Ethical Review Panel. Spleen, liver and mesenteric lymph node samples were collected from a healthy, 3-year-old male, Large White × Landrace pig, euthanised by intramuscular injection of ketamine followed by captive bolt. Total RNA was prepared using Qiagen RNeasy kit (Qiagen, Crawley, UK) according to manufacturer’s instructions, including a DNase digestion step. Porcine specific cDNA was produced using 1ug of total RNA and reversed transcribed using ImPromII (Promega, Southampton, UK). Successful cDNA production without genomic DNA contamination was demonstrated using porcine HPRT primers [38].

### 2.3 Expression cloning of porcine CSF-1 and CSF-1R

For mammalian expression of porcine CSF-1 and CSF-1R, PCR primer pairs (Table 1) were designed for amplification of biologically active porcine CSF-1 (amino acids 1–149) and full-length CSF-1R from the predicted porcine CSF-1 cDNA sequence (Pre-Ensemble BLA_hbFhA8F3M), and based on homologous regions of human, mouse and bovine CSF-1R for porcine CSF-1R, since the corresponding porcine genomic sequence was not available at the time of primer design.

Amplification was achieved using porcine cDNA and expand high fidelity enzyme (Roche, Mannheim, Germany) with 3 mM MgCl_2_ (CSF-1R) or 1 mM MgCl_2_ (CSF-1) using an initial cycle of 94 °C for 2 min (3 min for CSF-1R), followed by 30 cycles (35 for CSF-1R) of 94 °C for 30 s, 60 °C for 30 s (56 °C for CSF-1R), 72 °C for 3 min and one cycle of 72 °C for 10 min. PCR products were gel-purified using QIAquick gel extraction kit (Qiagen) and cloned in frame with V5-His C-terminal tag of pEF6/V5-His expression construct using TOPO cloning kit (Invitrogen). DNA sequencing was performed by DNA Sequencing & Services (MRCPPU, College of Life Sciences, University of Dundee, Scotland, www.dnaseq.co.uk) using Applied Biosystems Big-Dye Ver 3.1 chemistry on an Applied Biosystems model 3730 automated capillary DNA sequencer.

For bacterial expression of porcine CSF-1, the sequence corresponding to the active fragment of porcine CSF-1 (Ser36–Arg189) was codon optimised for expression in *Escherichia coli* and synthesized by Blue Heron Biotechnologies (WA, USA). The sequence was engineered with a *Bsp*HI restriction site at the 5′ end and an *Eco*RI restriction site at the 3′ end and cloned into the expression plasmid pET-28(b) using the complimentary restriction sites *Nco*I and *Eco*RI. The resulting plasmid, pTLW53, was transformed into MAX Efficiency® DH5α™ Chemically Competent *E. coli* according to the manufacturer’s protocol (Invitrogen, CA, USA). A kanamycin resistant transformant was selected and the plasmid sequenced to verify the error-free ORF. The pTLW53 plasmid was isolated via QIAprep® spin miniprep kit (Qiagen, CA, USA) according to the manufacturer’s recommendations and transformed into One Shot® BL21 Star™ Chemically Competent *E. coli* (Invitrogen, CA, USA).

An overnight TB/Kan^50^ broth of pTLW53/One Shot® BL21 Star™ *E. coli* incubating at 37 °C with 225 rpm shaking was refreshed 1:10 into 1 l of TB/Kan^50^ broth into baffled, vented 2L flasks. Protein expression was induced with 0.5 mM IPTG, final concentration, with incubation conditions continued at 37 °C and 300 rpm shaking. After 2 h induction, the culture was centrifuged and the *E. coli* pellet was stored at −80 °C.

Frozen cell pellets from *E. coli* cell culture were suspended in five volumes of lysis buffer (50 mM Tris pH 8.5, 5 mM EDTA) and lysis was completed by passing the suspension through a Microfluidizer. Lysate was centrifuged and insoluble pellets were washed in 1% Trition X-100, and 5 mM EDTA. Inclusion body pellets were suspended in DEAE buffer (15 mM Tris pH 8.5, 8 M Urea, 10 mM DTT, 1 mM EDTA), and mixed at room temperature for 60 min. Following clarification, the soluble protein was loaded onto a DEAE Sepharose column and eluted with a gradient of 0–150 mM NaCl in buffer containing 8 M Urea. Protein fractions containing pCSF-1 were pooled and diluted slowly into two parts redox buffer (50 mM Tris pH 8.5, 5 mM EDTA, 1 mM reduced glutathione, 1 mM oxidised glutathione). Protein was dialysed against redox buffer overnight and dialysis buffer exchanged to contain 0.5 mM reduced glutathione and 1 mM oxidised glutathione. Refolded pCSF-1 dimer was loaded onto a Q Sepharose column equilibrated with 50 mM Tris pH 8.5, 5 mM EDTA. Protein was eluted with a 10 BV gradient of 0–250 mM NaCl. The pooled pCSF-1 was dialysed against PBS and sterile filtered prior to use. Protein concentration was calculated by UV absorbance at 280 nm.

### 2.4 Generation of stable cell lines

For generation of stable Ba/F3 cells expressing porcine CSF-1R, 5 × 10^6^ Ba/F3 cells were transfected by electroporation (300 V, 975 μF) with 10 μg DNA (pEF6_pCSF-1R or empty pEF6 DNA), or no DNA, and selected with 30 μg/ml blasticidin (Invitrogen) and 10% IL-3 for 6 days prior to further selection with 30 μg/ml blasticidin and 10^4^ Units/ml of rh-CSF-1. For generation of stable pEF6_pCSF-1 HEK293T cells, 0.8 × 10^6^ cells/well of a six well plate were plated with antibiotic-free DMEM for 24 h, followed by transfection with 4 μg DNA (pEF6_pCSF-1, or empty pEF6 DNA), or no DNA, using Lipofectamine 2000 (Invitrogen) according to manufacturer’s instructions. Selection of stable cells was achieved by the addition of 10 μg/ml blasticidin (Invitrogen) after 48 h.

### 2.5 Immunoblotting

Whole cell lysate was prepared by lysing 0.5 × 10^6^ cells in 2% SDS 10 mM Tris buffer and boiling for 10 min at 100 °C. 100 μl of centrifuged supernatant from stably transfected HEK293T cell cultures of pEF6_pCSF-1, and empty pEF6 was also prepared. Protein concentration was determined using DC protein assay (Bio-Rad, Hercules, CA, USA) with 10 μg of protein mixed with Laemmli buffer (Invitrogen) and 5 mM DTT. Samples were run on a 4–12% gradient precast SDS–PAGE gel (Bio-Rad) and transferred onto polyvinylidene difluoride membrane, as per manufacturer’s directions (Bio-Rad). The membrane was blocked with 5% skimmed milk powder in TBS-Tween 20 at 4 °C overnight, prior to being washed and probed with 1:5000 dilution of mouse anti-v5 tag antibody (MCA1360G, AbD Serotec, Raleigh, NC, USA) and 1:5000 dilution of anti-mouse IgG HRP conjugated antibody (7076, Cell Signalling Technology, Beverly, USA,) and detected using enhanced chemiluminescence (ECL) reagents (Amersham, GE Healthcare, UK). β-Actin (Santa Cruz Biotechnology INC, sc-4778) was used at 1:200 dilution as a loading control.

### 2.6 Bone marrow differentiation

Pig bone marrow cells were obtained by flushing the bone marrow from five caudal ribs with 20 ml of complete RPMI with 5 mM EDTA using a bone marrow biopsy/aspirate needle (Cardinal Health, USA). For each condition, 0.25 × 10^6^ cells were pelleted and re-suspended in 4 ml of complete RPMI containing supernatant from empty pEF6 or pEF6_pCSF-1 transfected HEK293T cells (100%, 80%, 50% and 20%). Cells were plated into 60 mm bacteriological plates and incubated for 10 days at 37 °C with 5% CO_2_.

With owners consent, 0.5 ml of surplus feline bone marrow from clinical diagnostic investigation of a 2 year old, neutered male, DSH cat was obtained from a bone marrow aspirate of the left humerus using a bone marrow biopsy/aspirate needle (Cardinal Health, USA) and placed in EDTA. Canine bone marrow was collected post-mortem with owner’s consent from a 3-year-old male entire Staffordshire Bull Terrier dog which was euthanised for behavioural reasons. Bone marrow was flushed from the left femur with 20 ml of RPMI and 5 mM EDTA using a bone marrow biopsy/aspirate needle (Cardinal Health, USA). For both feline and canine bone marrow cultures, 10 × 10^6^ cells were cultured in 60 mm bacteriological plates with 4 ml RPMI, supplemented with either 10^4^ Units/ml rh-CSF-1, or 300 ng/ml porcine CSF-1 and incubated for 12 days at 37 °C with 5% CO_2_.

Mouse bone marrow cells were flushed from the femurs of an 8-week-old male BALB/c mouse with 10 ml complete RPMI and a 27 g needle, and plated in 100 mm bacteriological plates with complete RPMI supplemented with 10^4^ Units/ml rh-CSF-1 for 5 days at 37 °C with 5% CO_2_.

### 2.7 Cell viability assays

Stable Ba/F3 cells expressing porcine CSF-1R were maintained in culture with complete RPMI supplemented with either 10^4^ Units/ml rh-CSF-1 or 10% IL-3 conditioned medium prior to MTT assay. 2 × 10^4^ cells/well (Ba/F3 cells and Ba/F3 transfectants), or 5 × 10^4^ cells/well (mouse BMM) of a 96 well plate were plated in triplicate or quadruplicate and appropriate treatment (serial dilutions of rh-CSF-1, rm-CSF-1 (R&D systems 416-ml) rhIL-34 (R&D Systems 5265), or rmIL-34 (R&D Systems 5195), or supernatant from pEF6_pCSF-1 were added to make a total volume of 100 μl per well. Cells were incubated for 48 h at 37 °C with 5% CO_2_. For Ba/F3 cells, 10 μl of MTT (Sigma Aldrich M5655) stock solution (5 mg/ml) was added directly to each well to achieve a final concentration of 0.5 mg/ml and incubated at 37 °C for 3 h prior to solubilisation overnight. For adherent mouse BMM cells, culture medium was replaced with 50 μl of 1 mg/ml MTT solution and incubated for 1 h at 37 °C. MTT solution was removed and tetrazolium salt solubilised with 100 μl of solubilisation agent (0.1 M HCl, 10% Triton X-100 and isopropanol) followed by incubation at 37 °C with 5% CO_2_ for 10 min. Plates were read at 570 nm with reference wavelength of 405 nm.

### 2.8 EC_50_ calculation

The EC_50_ for rh-CSF-1 and bacterially produced porcine CSF-1 in the Ba/F3pCSF-1R MTT cell viability assay were calculated from dose responses performed in triplicate or quadruplicate. Data was analysed using GraphPad Prism software using nonlinear regression curve fit. The *R*^2^ (coefficient of determination) values for all curve fits were >0.90.

### 2.9 3D modelling of contact amino acids

3D models in PDB format were generated with 3D-Jigsaw (http://bmm.cancerresearchuk.org/3djigsaw/) using structure-based alignments (performed by Domain Fishing). 3D models of human IL-34-CSF-1R (PDB 4DKD), mouse IL-34-CSF-1R (PDB 4EXP), and mouse CSF-1 (PDB 3EJJ) were obtained and viewed in FirstGlance in Jmol (http://firstglance.jmol.org). Human and pig CSF-1 were generated using 3D-Jigsaw with the mouse CSF-1 structure as template (3EJJ) and pig CSF-1R was generated using human CSF-1R as template (4DKD chain X). Contact amino acids between IL-34, CSF-1 and CSF-1R were identified using recently published data [5,21,22]. The non-conserved contact amino acids of human and mouse IL-34, CSF-1 and CSF-1R were highlighted.

## 3 Results

### 3.1 Cloning and sequence of porcine CSF-1 and CSF-1R

**Fig. 1:**
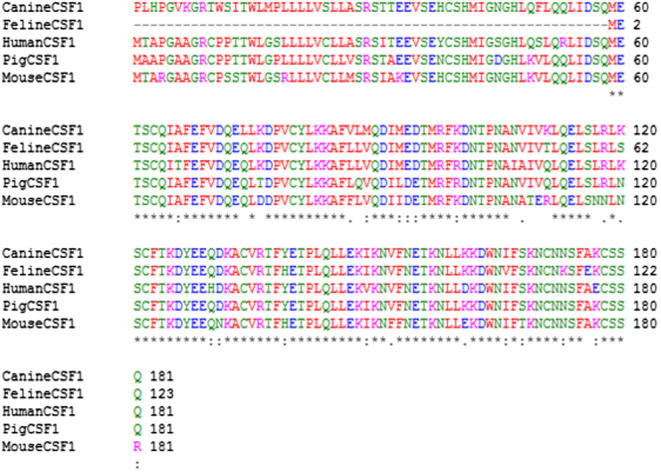
Alignment of cloned porcine CSF-1 with human, mouse, canine and feline CSF-1. Alignment was performed using Clustal W (http://www.ebi.ac.uk/Tools/msa/clustalw2/) and demonstrates the high level of homology that exists between porcine and human CSF-1. The red colour represents small hydrophobic amino acids, blue colour represents acidic amino acids, magenta denotes basic amino acids, and the green colour corresponds to hydrophilic or polar amino acids. Identical amino acids are represented by “∗”, conserved substitutions are represented by “:” and semi-conserved substitutions are represented by “.”. (Note: signal peptide amino acid sequence is missing from feline CSF-1).

A partial fragment of porcine CSF-1 cDNA was cloned previously and used to demonstrate the existence of multiple isoforms, similar to those in humans, in conceptus and uterine tissues of the pig [10]. However, the sequence was not reported. For our study, porcine CSF-1 cDNA was PCR-amplified from liver, mesenteric lymph node and spleen cDNA templates. Agarose gel electrophoresis of the PCR products revealed the expected single band size of approximately 550 base pairs in all three-tissue samples, with the spleen sample producing the most product (data not shown). For CSF-1R, PCR amplification using porcine spleen cDNA template identified the expected single band size of approximately 3000 base pairs. The coding regions for porcine CSF-1 and CSF-1R were cloned in frame with V5-His C-terminal tag of pEF6 V5-His TOPO plasmid. The DNA and protein sequences were confirmed by sequence analysis. Multiple species alignments of CSF-1 and CSF-1R were consistent with the published porcine CSF-1, and CSF-1R sequences (ENSEMBL ENSSSCT00000007466 for CSF-1 and ENSSSCP00000015371 for CSF-1R). The cloned porcine CSF-1 shares 99% amino acid homology with the reference genomic sequence in ENSEMBL. A single nucleotide change in the signal peptide was associated with a single amino acid change at position 2 of the CSF-1 signal peptide from Thr to Ala. This mutation may be due to PCR proof-reading error rather than single nucleotide polymorphism (SNP) since other species (cow, human, mouse, guinea pig and rat) all have Thr present at amino acid 2. The cloned porcine CSF-1 cDNA sequence is 543 base pairs in length and encodes a 32 amino acid signal peptide (Met1–Ala32) and a 149 amino acid mature protein in which the biological activity is predicted to be maintained (Glu33–Ser190). The porcine CSF-1 peptide sequence has 87% and 84% homology with human and mouse CSF-1 amino acid sequences respectively, while human and mouse share 80% homology (Fig. 1). The seven Cys residues that form the three intra-chain disulphide bonds and the single inter-chain disulphide bond required for biological activity [39] are conserved. The residues involved in the binding of porcine CSF-1 to CSF-1R are shown in Table 2 together with mouse and human data [5].

**Fig. 2:**
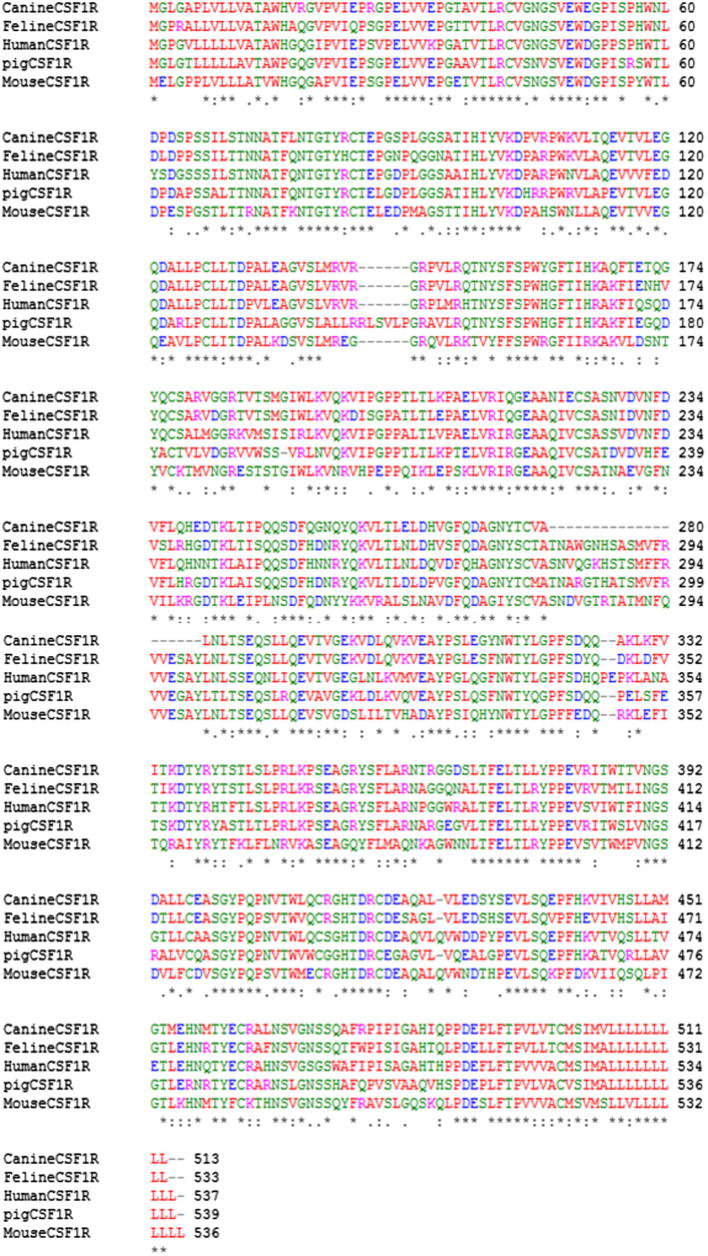
Alignment of the cloned porcine CSF-1R extracellular domain with human, mouse, canine and feline CSF-1R. Alignment was performed using Clustal W (http://www.ebi.ac.uk/Tools/msa/clustalw2/) and demonstrates the high level of homology that exists between porcine and human CSF-1R. The red colour represents small hydrophobic amino acids, blue colour represents acidic amino acids, magenta denotes basic amino acids, and the green colour corresponds to hydrophilic or polar amino acids. Identical amino acids are represented by “∗”, conserved substitutions are represented by “:” and semi-conserved substitutions are represented by “.”.

The cloned porcine CSF-1R is 2904 base pairs in length which encodes full-length CSF-1R of 969 amino acids, including a 19 amino acid signal peptide (Met1–Gly19) and a 950 amino acid mature chain (Val20–Cys969). The 520 amino acid extracellular segment contains the predicted ligand binding domains (D1–D5) and a 25 amino acid hydrophobic transmembrane region. The 430 amino acid intracellular region contains the tyrosine kinase domain and ATP binding region required for catalytic activity of the receptor upon binding of CSF-1 or IL-34 to the extracellular domain. Comparison between species of CSF-1R sequences demonstrates that porcine CSF-1R shares 80% homology with human CSF-1R and 72% homology with the mouse CSF-1R amino acid sequence, while human and mouse CSF-1R share 75% amino acid homology (Fig. 2). In particular, conserved regions of amino acids are noted within the predicted binding sites for CSF-1 (D2–D3 immunoglobulin domain) and IL-34 (D3–D4 interface region of the extracellular domain) in human and pig CSF-1R. Further homology also exists between the signal peptide, transmembrane region and ATP binding site of the intracellular domain. The ten cysteine residues present in the extracellular domain that are most likely responsible for the tertiary structure of CSF-1R are conserved between human, mouse and the cloned porcine CSF-1R. The presence of these residues is a feature shared by other type III receptor kinases family members e.g. cKIT and PDGF, thus highlighting the evolutionary origins of these receptor family members [40]. The five tyrosine residues (Tyr546, Tyr699, Tyr708, Tyr723, Tyr809 and Tyr923) located within the CSF-1R intracellular domain, which are phosphorylated in response to CSF-1 binding, thus activating downstream signalling, are also conserved between human, mouse and porcine CSF-1R. Based on this high level of conservation between human and porcine CSF-1R and CSF-1, we predicted that human CSF-1 would bind and activate the porcine CSF-1R.

### 3.2 Production of porcine CSF-1 from HEK293T cells

**Fig. 3:**
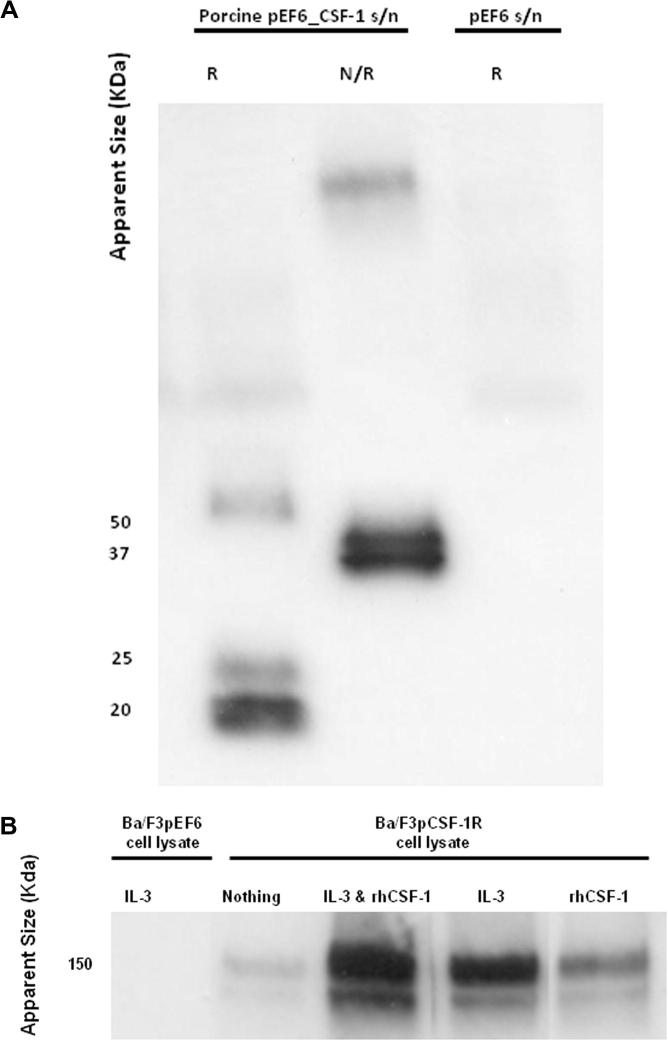
Expression of cloned porcine CSF-1 (A) and CSF-1R (B). (A) Western blot of secreted cloned porcine CSF-1 by HEK293T cells transfected with porcine CSF-1_pEF6 expression construct or empty pEF6 construct. Under non-reducing conditions, two bands of approximately 37 KDa and 50 KDa were detected, whereas two smaller bands (20 and 25 KDa) were detected in the presence of dithiothreitol (DTT). Porcine CSF-1 is secreted as a disulphide linked dimer that is variably glycosylated. (B) Western blot of cloned expressed porcine CSF-1R transfected into Ba/F3 cells. Cells were cultured in either rh-CSF-1, IL-3 or both factors combined prior to collection of cell lysate. Differential levels of receptor expression were noted when the cells were cultured in these different conditions. Receptor expression is reduced when Ba/F3 cells expressing CSF-1R are cultured in rh-CSF-1 alone.

To provide an initial source of porcine CSF-1 for *in vitro* studies, the pEF6_CSF-1 expression plasmid encoding porcine CSF-1 was transfected into HEK293T cells using Lipofectamine 2000. Secreted CSF-1 in the HEK293T supernatant was successfully detected using Western blotting for the V5 epitope tag encoded by the expression plasmid. The CSF-1 protein has a predicted weight of 27 KDa. Under non-reducing conditions, two bands of approximately 37 KDa and 50 KDa were detected, whereas two smaller bands (20 and 25 KDa) were detected in the presence of dithiothreitol (DTT) (Fig. 3A). These findings suggest that the recombinant protein is expressed and secreted as a disulphide-linked dimer, and may be variably glycosylated.

### 3.3 Production of porcine CSF-1R expressing Ba/F3 cells

To confirm biological activity of secreted porcine pEF6_CSF-1, we stably transfected the IL-3 dependant Ba/F3 cell line [41], as previously described for the human receptor [31], with porcine CSF-1R. Stable clones were initially selected for their survival in blasticidin, followed by further selection in rh-CSF-1. The expression of porcine CSF-1R on Ba/F3 cells abrogated the absolute IL-3 dependance of the Ba/F3 cells and permitted a proliferative response in response to CSF-1. Parent Ba/F3 cells and Ba/F3 cells transfected with the empty expression construct (pEF6) which did not express CSF-1R did not survive in the presence of rh-CSF-1 upon removal of IL-3.

Western blot analysis of these cells based upon detection of the V5 epitope tag demonstrated successful expression of porcine CSF-1R. Prior selection of these cells with rh-CSF-1 and/or IL-3 altered the levels of receptor expression (Fig. 3B). Growth of Ba/F3 cells expressing porcine CSF-1R in rh-CSF-1 reduced levels of receptor expression compared to cells grown in the presence of IL-3 or both growth factors combined.

### 3.4 Activation of porcine CSF-1R with CSF-1

**Fig. 4:**
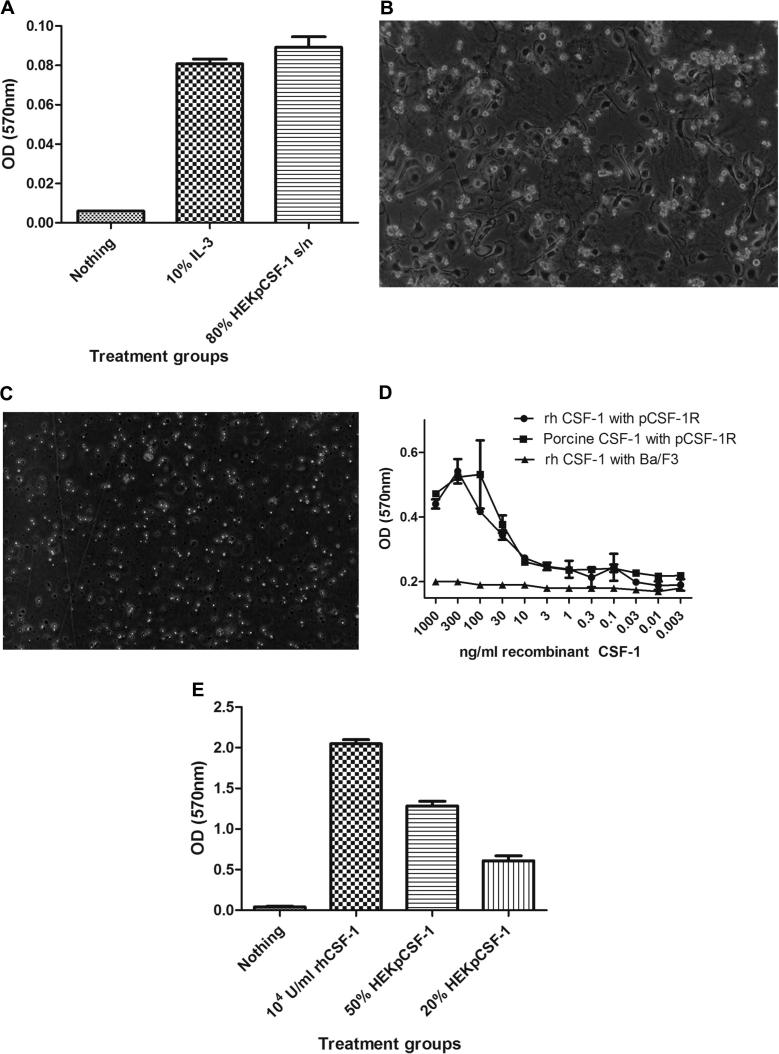
Demonstration of biological activity of cloned secreted porcine CSF-1 transfected into HEK293T cells and porcine CSF-1 expressed in *E.Coli*. (A) An MTT cell viability assay was performed using Ba/F3 cells expressing porcine CSF-1R and supernatant collected from transfected HEK293T cells with porcine CSF-1_pEF6 expression construct. Using 80% of the HEK293T transfected cell supernatant produced viable cells. (B) Porcine bone marrow cells cultured with 20% supernatant collected from HEK293T cells transfected with porcine CSF-1_pEF6 expression construct differentiated into BMDMs, adhered to the tissue culture plate and proliferated compared to cells cultured with supernatant from HEK293T cells transfected with empty pEF6 construct which did not adhere, proliferate or survive after 7 days in culture (C). (D) Bacterially expressed Porcine CSF-1 has also shown to be biologically active on the porcine CSF-1R expressed in Ba/F3 cells in an MTT cell viability assay. (E) An MTT cell viability assay was performed using mouse BMMs cultured with supernatant collected from transfected HEK293T cells with porcine CSF-1_pEF6 expression construct. Using either 50% and 20% supernatant produced viable cells. All of the assays shown are representative of three separate experiments with either triplicate or quadruplicate determinations.

Using an MTT cell viability assay, we developed and optimised a bioassay for assessing the biological activity of secreted CSF-1 by HEK293T transfection with porcine pEF6_CSF-1, and rh-CSF-1. The bioassay was optimised for cell number/well, and culture conditions (rh-CSF-1, and/or IL-3) prior to assay, but these variables did not make a substantive difference to sensitivity. Although IL-3 was equivalent to rh-CSF-1 in the bioassay in terms of cellular viability and proliferative response of the Ba/F3 cells expressing CSF-1R, rh-CSF-1 was used as the standard culture growth factor to maintain selection pressure for CSF-1R expression. Using the MTT cell viability assay, the presence of 10% conditioned medium from the IL-3-expressing cell line allowed survival of both parent Ba/F3 and Ba/F3 cells expressing porcine CSF-1R. The supernatant from transfected HEK293T with porcine pEF6_pCSF-1 also maintained the viability of the Ba/F3 cells expressing porcine CSF-1R (Fig. 4A).

Bone marrow-derived macrophages (BMDMs) grown in CSF-1 have been used extensively in studies of mouse macrophage biology [42,43]. We therefore tested whether porcine CSF-1 in the HEK293T cell supernatant would be active on the porcine CSF-1R in its native context by causing bone marrow progenitor cells to differentiate into BMDMs. Porcine bone marrow progenitor cells isolated from a rib, cultured for 7 days in either 100%, 80%, 50% or 20% HEK293T pEF6_pCSF-1 supernatant, grew and differentiated into adherent macrophages (Fig. 4B) whereas control cells died (Fig. 4C). We describe elsewhere the use of pig BMDM grown in recombinant human CSF-1 in studies of macrophage responses to bacterial lipopolysaccharide [44]. In combination, these experiments have confirmed both functionality of the expressed porcine CSF-1R and biological activity of HEK293T pEF6_pCSF-1 supernatant on both our CSF-1R expressing cell line, and porcine primary cells.

### 3.5 Expression of porcine CSF-1 in bacteria

The recombinant human CSF-1 used here and in previous studies on pig marrow [44] was expressed in *E. coli*. The advantage of an *E. coli* expression system is not only the high yield which enables both preclinical and structural studies, but also the possibility of introducing defined mutations to support structure–function analysis. To enable such studies of the porcine protein, and to further optimise expression, a synthetic codon-optimised cDNA encoding the active amino acids was generated as described in Section 2. The EC_50_ of the bacterially produced porcine CSF-1 and rh-CSF-1 on Ba/F3 cells expressing porcine CSF-1R was essentially identical (29 ng/ml and 34 ng/ml respectively) (Fig. 4D).

### 3.6 Species specificity of CSF-1 and IL-34

**Fig. 5:**
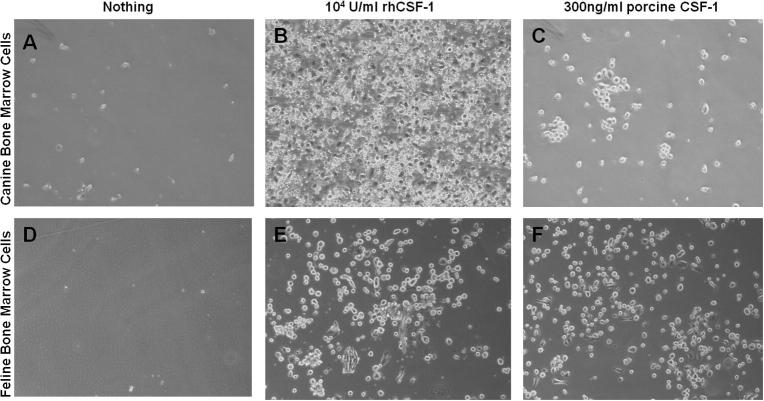
Activity of porcine and human recombinant CSF-1 on canine and feline bone marrow progenitor cells. Canine and feline bone marrow cells were cultured with either 10^4^ U/ml rh-CSF-1, 300ng/ml porcine *E.Coli* expressed CSF-1 or no growth factors. By day 5 of differentiation, canine and feline BMDMs with no growth factors were dead (canine A & feline D). By day 5 of differentiation for canine and day 12 for feline, BMDMS were attaching to the culture dish when cultured with either rh-CSF-1 (canine B & feline E) or porcine CSF-1 (canine C & feline F).

Human CSF-1 is active on mouse CSF-1R, but not vice versa [28,45,46]. Mouse CSF-1 expressed by L929 fibroblasts is biologically active on the porcine CSF-1R expressed on peripheral blood mononuclear cells and bone marrow progenitor cells [47,48], but the precise efficacy was not previously determined. Using an MTT cell viability assay we demonstrated that recombinant mouse and human CSF-1 also have identical activity on the porcine CSF-1R. The pig provides an apparent intermediate between the species, enabling analysis of the molecular basis of the species specificity. We therefore assessed the biological activity of porcine CSF-1 on the mouse CSF-1R. Using an MTT cell viability assay, mouse BMMs were cultured in the presence of 10^4^ Units/ml rh-CSF-1 or increasing concentrations of HEK pEF6_pCSF-1 supernatant. There was a dose-dependent increase in BMM survival with increasing concentrations of HEK pEF6_pCSF-1 supernatant (Fig. 4E). This finding was extended by demonstrating that both human and porcine CSF-1 have similar biologically activity on bone marrow cells isolated from the cat and dog. Bone marrow progenitor cells from both species proliferated, became adherent and differentiated into a mature population of bone marrow derived macrophages (BMDM) (Fig. 5). Hence, porcine CSF-1, like human CSF-1, is biologically active in all mammal species tested. The species barrier is restricted to the relative lack of activity of the mouse protein on human cells.

### 3.7 Activation of porcine CSF-1R with IL-34

**Fig. 6:**
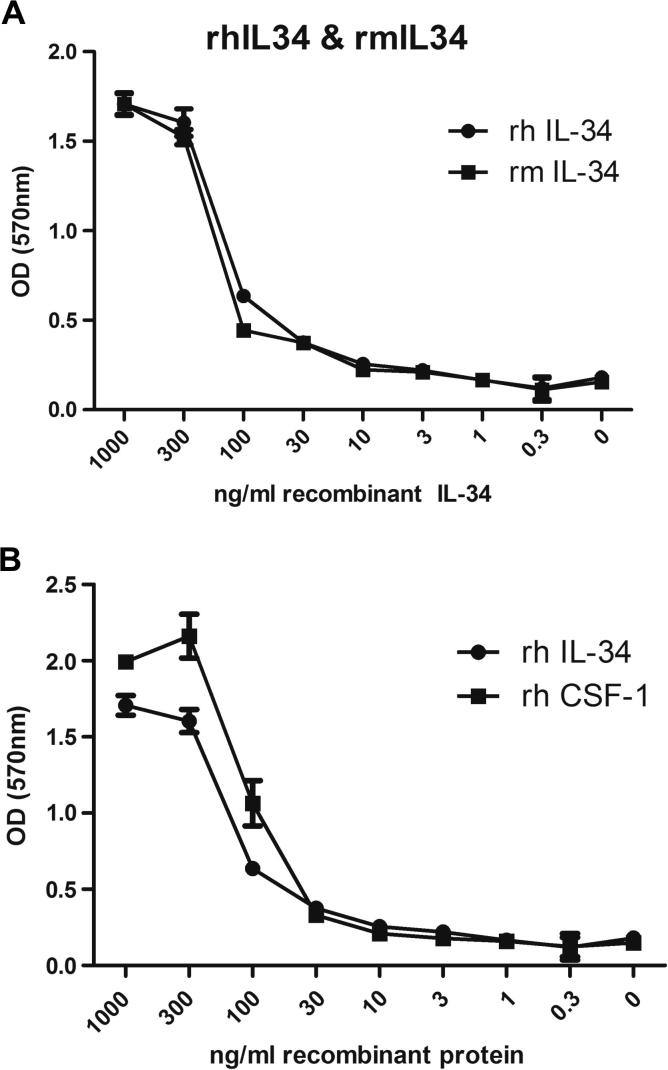
Activity of recombinant human and mouse IL-34 on porcine CSF-1R expressed in Ba/F3 cells. An MTT cell viability was used to assess the biological activity of human and mouse IL-34 on expressed porcine CSF-1R. (A) Both human and mouse IL-34 are biologically active on the porcine receptor. (B) Human IL-34 has demonstrates similar activity to rh-CSF-1 on the porcine CSF-1R in an MTT assay.

Previous studies suggest that CSF-1 and IL-34 bind different parts of the CSF-1R [12], but with similar outcomes in terms of the survival and proliferation of human blood monocytes and colony formation from bone marrow progenitors [19]. In contrast to CSF-1, human IL-34 was found to be considerably less active than the mouse protein in stimulating CSF-1R mediated mouse macrophage proliferation [49], a finding we have confirmed (not shown). Recombinant mouse and human IL-34 are both commercially available (R&D Systems). Both products are the more active isoforms that include an indel (Q81) generated by an alternative splice acceptor [49], which is also present in the pig IL-34 gene (ENSEMBL ENSSSCP00000002942). We therefore tested their comparative efficacy on the Ba/F3 cells expressing porcine CSF-1R (Fig. 6A). Mouse and human IL-34 were almost equally active. We also compared the efficacy of human CSF-1 and IL-34. Human IL-34 was found to have similar activity to rh-CSF-1 on the porcine CSF-1R (rhIL-34 EC_50_ 137 ng/ml and rhCSF-1 EC_50_ 100 ng/ml) (Fig. 6B). This finding indicates that pig is an intermediate species in which to test therapeutic applications of recombinant human IL-34. Surprisingly, and despite the fact that we have succeeded in expressing avian IL-34 in transfected mammalian cells [12] we have not yet been able to produce biologically active pig IL-34 through mammalian expression.

## 4 Discussion

We have produced a stable Ba/F3 cell line expressing porcine CSF-1R which can be utilised in a cell viability bioassay to assess biological activity of CSF-1. Numerous receptors and oncogenes have been tested in Ba/F3 cells for their ability to generate factor-independent, or novel factor-dependent growth [31,50]. An earlier study reported their stable transfection with human CSF-1R, or the related flt3 or c-kit receptor, to develop assays for specific inhibitors [31]. In that study, Ba/F3 cells were stably-transfected with the human CSF-1R expression construct containing a blasticidin resistance cassette, selected in blasticidin, then subjected to secondary selection for the ability to grow in rh-CSF-1 in the absence of IL-3. This approach generated stable transfectants with both the human receptor and a mouse-human chimeric receptor [31]. We have successfully reproduced this approach using porcine CSF-1R and then selected cells for their ability to grow in rh-CSF-1.

Although transfected Ba/F3 cells grew well in CSF-1 and provided a useful assay, they nevertheless generally proliferated less rapidly than in IL-3. Ba/F3 cells were originally identified as pro-B cells that express surface Ig and lack endogenous CSF-1R [41,51,52]. However, although we have confirmed the presence of surface Ig, we find that they express the myeloid markers Cd11b and F4/80 antigens (data not shown). In this respect, they share some features with other myeloid lines such as PU5/1.8 and P388D1 [53]. The addition of CSF-1 to the receptor-expressing cells apparently induces some of them to undergo differentiation, growth arrest and adhesion. Western blotting of the Ba/F3 cells expressing porcine CSF-1R revealed differential levels of receptor expression, depending on the culture conditions. When cells were cultured in rh-CSF-1, there was a reduction in the level of CSF-1R expression. This result may be explained by ligand-receptor activation followed by degradation and internalisation of the CSF-1/CSF-1R complex [54,55]. The ability of IL-3 to prevent the loss of CSF-1R was unexpected; it may be due to IL-3 producing a signal that inhibits CSF-1/CSF-1R interactions, degradation of the CSF-1R after binding of CSF-1, or CSF-1R internalisation. An alternative explanation is that IL-3 acts on the EF1A promoter in the expression plasmid to increase CSF-1R production.

**Fig. 7:**
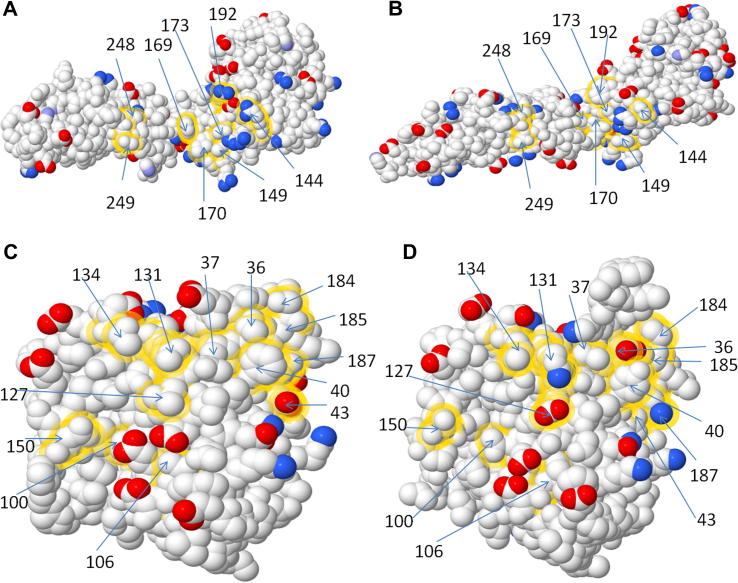
3D models of non-conserved contact amino acids between human and mouse CSF-1R and IL-34. 3D models demonstrating the charged amino acid changes of human and mouse CSF-1R and IL-34 were generated using the PDB file 4DKD (human) and 4EXP (mouse). Published contact amino acids for both human and mouse CSF-1R and IL-34 were analysed and non-conserved contact amino acids highlighted using FirstGlance (http://firstglance.jmol.org). (A) 3D model of human CSF-1R non-conserved amino acids for IL-34 binding and (B) mouse CSF-1R non-conserved amino acids for IL-34 binding. (C) 3D model of human IL-34 non-conserved contact amino acids and (D) 3D model of mouse IL-34 non-conserved contact amino acids. Positively charged atoms are represented by blue colour and negatively charged atoms by red colour. Medium blue coloured atoms denote partially charged atoms.

We have used the recombinant proteins and receptor-expressing cells to further dissect the species-specificity of CSF-1 and IL-34. Porcine CSF-1 was able to activate porcine, mouse, feline and canine CSF-1 receptors, a property shared by recombinant human CSF-1 [28,48,56,57]. Conversely, mouse CSF-1 which lacks activity on human cells [46,47,58] was able to activate the porcine receptor, and mouse and human IL-34, which lacks cross-reactivity across those two species [19], were both active on the porcine receptor. The contact amino acids between human CSF-1R and IL-34 has recently been published [22]. Logically, the CSF-1R from the three species must differ from each other in contact amino acids with IL-34. Fig. 7A and B shows the models of human and mouse CSF-1R viewed from the perspective of IL-34, highlighting the contact amino acids that are not conserved between human and mouse. The sequence alignment in Fig. 2 shows that the contiguous binding patch from amino acid 169–173 is VLDSNT in mouse CSF-1R, FIEGQD in pig CSF-1R and FIQSQD in human CSF-1R. CSF-1R contact amino acids 248 and 249 are Pro and Leu in mouse, Ser and Gln in pig and Pro and Gln in human. A third significant difference between the species exists around amino acid 144–150 of the CSF-1R. Gly103 in IL-34, which is conserved in mouse, human and pig, forms a salt bridge with Arg142 and Arg146 in human CSF-1R (22). In mouse, the sequence is REGGR, in human it is RVRGR and in pig, there is a complex insertion of six amino acids relative to all other mammalian CSF-1R (LLRRLSVLPGR) (Fig. 2). The amino acid differences between the receptors from mouse and human also cause subtle changes in the predicted topology, as shown in Fig. 7A and B. If we consider the ligand IL-34, Fig. 7C and D shows the binding faces of human and mouse ligands, again highlighting the non-conserved contact amino acids. Despite the substantial conservation across species, and low D_N_/D_S_ ratios, noted by Garceau et al. [12], the majority of the amino acids shown are divergent between mouse, pig and humans. For example, amino acids 127–134 are NVQQGLTD in human, DVRQGLAG in pig and NVQRSLMD in mice. Accordingly, there is no simple model to explain the species specificity of IL-34 actions and despite the apparent overall conservation of IL-34 [12], the contact amino acids are under positive selection, consistent with a role in the immune system. Since the cat and dog CSF-1R also differ from each other (Fig. 2), and from mouse, human and pig, in the key contact amino acids, cross-species reactivity of IL-34 will probably need to be determined empirically.

**Fig. 8:**
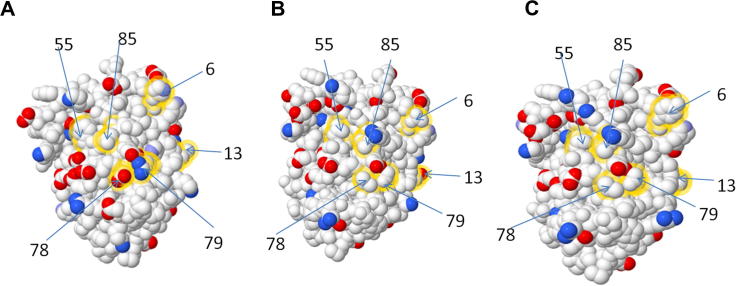
3D models of non-conserved contact amino acids between human and mouse and porcine CSF-1 and CSF-1R. 3D models demonstrating the charged amino acid changes of human, porcine and mouse CSF-1 were generated using the PDB file for mouse CSF-1 (3EJJ) was used as a template for both human and porcine CSF-1 models. Published contact amino acids of mouse CSF-1 were analysed and non-conserved contact amino acids highlighted using FirstGlance (http://firstglance.jmol.org). (A) 3D model of mouse CSF-1 non-conserved contact amino acids, (B) 3D model of porcine CSF-1 non-conserved contact amino acids and (C) human CSF-1 non-conserved contact amino acids. Positively charged atoms are represented by blue colour and negatively charged atoms by red colour. Medium blue coloured atoms denotes partially charged atoms.

**Fig. 9:**
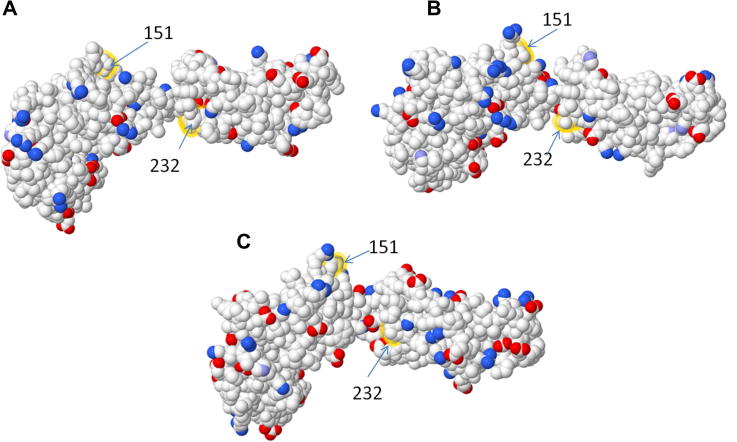
3D models of non-conserved contact amino acids between mouse, human and porcine CSF-1 binding sites of CSF-1R. 3D models highlighting the non-conserved CSF-1 contact binding sites of mouse, human and porcine CSF-1R. Human and mouse CSF-1R models were generated using the PDB file 4DKD (human) and 4EXP (mouse). Porcine CSF-1R was generated using human CSF-1R (4DKD) as template. The 6 non-conserved amino acids involved in CSF-1 binding to CSF-1R (Glu78, Asn13, Asn85, His6, Phe55, Arg79) were identified and the coresponding binding site of CSF-1R identified (A) Mouse CSF-1R highlighting the position of Lys151 and Gly232. (B) Human CSF-1R and (C) Porcine CSF-1R highlighting these amino acids. Whilst the change from Lys to His on the human CSF-1R does not alter the amino acid properties, there is an increase in both the molecular and residue weight which could potentially constrain the binding of the mouse protein to the human receptor. The substitution of Gly232 in the mouse receptor with the larger Asn232 in human might produce a steric hindrance that is not seen when histidine is substituted as in the pig. Positively charged atoms are represented by blue colour and negatively charged atoms by red colour. Medium blue coloured atoms denotes partially charged atoms.

We have demonstrated that mouse CSF-1 can bind and activate the porcine CSF-1R. Porcine CSF-1 can also stimulate the mouse, dog and cat receptors. Conversely, mouse CSF-1 can activate porcine CSF-1R but not human CSF-1R. As with IL-34 binding, there must be a difference between all three species of CSF-1R and between mouse and human CSF-1. There are 19 contact amino acids between mouse CSF-1 and CSF-1R [5], 13 of which are conserved (Table 2). The remaining six amino acids (His6, Asn13, Phe55, Glu78, Arg79, Asn85 in mouse) are different in both pig and human. Fig. 8 highlights these non-conserved amino acids and their location on the binding surface of mouse (Fig. 8A), pig (Fig. 8B) and human (Fig. 8C) CSF-1. Of these, only 2 also have a corresponding change in the CSF-1R contact amino acid between all three species, His6 and Glu78. Glu78 of the mouse CSF-1 is Val78 in both the human and porcine CSF-1. Glu78 contacts Lys151 on the mouse CSF-1R, which is His151 in human CSF-1R. The bulkier amino acid at this position could constrain the binding of the mouse protein (Fig. 9A–C). In the pig, Lys151 is replaced with Gln, structurally similar but smaller and uncharged. The same substitution occurs in the cow, which can also respond to mouse CSF-1 [63]. His6 forms Van der Waals contacts with Gly232 in the mouse receptor. However, in this position, dog and cow share Asn232 with human, where pig has His232. So, this variation is less likely to determine binding specificity. Having established the bacterial expression system for the pig protein, structure function predictions can be tested in the future by targeted substitutions.

Mice with no detectable biologically-active serum CSF-1 (*op*/*op*), have been previously well studied [59]. Amongst other defects, these mice are growth retarded and are born with low birth weights compared to litter mate controls. We and others have explored therapeutic options for CSF-1 in a number of preclinical applications, mainly to do with tissue repair [33,60–62]. The successful expression of recombinant CSF-1 and CSF-1R in the pig and evaluation of cross-species activity of IL-34 provide the tools for evaluation of both agents in pig and rodent preclinical models.

## Figures and Tables

**Table 1:** Table of final primers used for cloning of porcine CSF-1, and CSF-1R. A Kozak sequence (bold in sequence) was included in both forward primers for optimal translation initiation (Kozak 1987).

Primer name	Primer sequence 5′–3′	*T_m_* (°C)	Size (bp)
Porcine CSF-1 forward	**AGTATGG**CCGCGCCGGG	61.3	543
Porcine CSF-1 reverse	CTGGCTGGAGCATTTAGCAAAGCT	59.9	–
Porcine CSF-1R forward	**ACCATGG**GCCTGGGCACGC	64.6	2904
Porcine CSF-1R reverse	GCAGAACTGGTAGGTGTTGGGTTGCAG	57.7	–

**Table 2:** Table of contact amino acids between mouse, human and porcine CSF-1 and CSF-1R.

CSF-1 mouse	CSF-1 pig	CSF-1 human	CSF-1R mouse	CSF-1R pig	CSF-1R human
**Asp59**	**Asp**	**Asp**	**Arg146**	**Arg**	**Arg**
Glu78	Val	Val	Lys151	Gln	His
Glu78	Val	Val	**Lys168**	**Lys**	**Lys**
**Met10**	**Met**	**Met**	**Tyr257**	**Tyr**	**Tyr**
Asn13	Asp	Ser	**Asp251**	**Asp**	**Asp**
**Gly14**	**Gly**	**Gly**	**Arg146**	**Arg**	**Arg**
**Gln58**	**Gln**	**Gln**	**Arg150**	**Arg**	**Arg**
**Asp62**	**Asp**	**Asp**	**Arg150**	**Arg**	**Arg**
Asn85	Leu	Leu	Leu170	Ile	Ile
His6	Asn	Tyr	**Val231** Gly232	**Val**, His	**Val**, Asn
**His9**	**His**	**His**	**Val231 Ser250 Tyr257**	**Val, Ser, Tyr**	**Val, Ser, Tyr**
**Met10**	**Met**	**Met**	**Val231 Tyr257**	**Val and Tyr**	**Val Tyr**
**Gly12**	**Gly**	**Gly**	**Asp251**	**Asp**	**Asp**
Asn13	Asp	Ser	**Asp251**	**Asp**	**Asp**
**Gly14**	**Gly**	**Gly**	**Asp251 Phe252**	**Asp, Phe**	**Asp, Phe**
**His15**	**His**	**His**	**Phe252 Tyr257**	**Phe, Tyr**	**Phe, Tyr**
Phe55	Leu	Leu	**Arg146**	**Arg**	**Arg**
**Gln58**	**Gln**	**Gln**	Arg142 **Arg146**	Leu, **Arg**	Arg, **Arg**
**Asp62**	**Asp**	**Asp**	Leu149 **Arg150**	Leu, **Arg**	Met, **Arg**
**Arg66**	**Arg**	**Arg**	**Arg150**	**Arg**	**Arg**
Arg79	Gln	Gln	**Phe252 Asn255**	**Phe, Asn**	**Phe, Asn**
Gln81	**Gln**	**Gln**	Leu149	Leu	Met
Glu82	**Glu**	**Glu**	Val169	Phe	Phe
Asn85	Leu	Leu	Leu170 Ser172 Asn173	Ile, Gly, Gln	Ile, Ser, Gln

Bold indicates conserved amino acids between these three species.
